# Determining anterior hip coverage in patients with hip dysplasia using the anterior center-edge angle on Lequesne’s false-profile radiograph and on computed tomography

**DOI:** 10.1093/jhps/hnac048

**Published:** 2023-03-06

**Authors:** Hui Cheng, Liqiang Zhang, Dianzhong Luo, Ningtao Ren, Zhendong Zhang, Wang Gu, Yongcheng Hu, Hong Zhang

**Affiliations:** Graduate School, Tianjin Medical University, No. 22, Qiaotai Road, Heping District, Tianjin, China; Senior Department of Orthopedics, The Fourth Medical Center of Chinese PLA General Hospital, No. 51, Fucheng Road, Haidian District, Beijing, China; Graduate School, Tianjin Medical University, No. 22, Qiaotai Road, Heping District, Tianjin, China; Department of Orthopaedics, Shanxi Children’s Hospital, No. 65, Jinxi Street, Jinyuan District, Shanxi, Taiyuan, China; Senior Department of Orthopedics, The Fourth Medical Center of Chinese PLA General Hospital, No. 51, Fucheng Road, Haidian District, Beijing, China; Senior Department of Orthopedics, The Fourth Medical Center of Chinese PLA General Hospital, No. 51, Fucheng Road, Haidian District, Beijing, China; Senior Department of Orthopedics, The Fourth Medical Center of Chinese PLA General Hospital, No. 51, Fucheng Road, Haidian District, Beijing, China; Senior Department of Orthopedics, The Fourth Medical Center of Chinese PLA General Hospital, No. 51, Fucheng Road, Haidian District, Beijing, China; Graduate School, Tianjin Medical University, No. 22, Qiaotai Road, Heping District, Tianjin, China; Department of Bone and Soft Tissue Oncology, Tianjin Hospital, No. 406, Jiefang South Road, Hexi District, Tianjin, China; Senior Department of Orthopedics, The Fourth Medical Center of Chinese PLA General Hospital, No. 51, Fucheng Road, Haidian District, Beijing, China

## Abstract

Anterior hip coverage is important for hip stability. As a parameter of anterior hip coverage, the anterior center-edge angle on false-profile radiograph (ACEA FP) is associated with clinical outcomes. With the widespread application of computed tomography (CT), the anterior center-edge angle on CT (ACEA CT) has also been used to measure anterior hip coverage. Little is known about the reproducibility of the ACEA FP and ACEA CT in patients with hip dysplasia or the correlation between the ACEA CT and ACEA FP. In total, 49 hips of 49 patients who underwent periacetabular osteotomy in our center were included. The lateral center-edge angle, Tönnis angle, ACEA FP and ACEA CT were determined. We assessed the intraobserver and interobserver reliability of the ACEA FP and ACEA CT, the effect of the Tönnis angle on the reliability of the ACEA FP and ACEA CT and the correlation between the ACEA CT and ACEA FP. The intraobserver and interobserver interclass correlation coefficients of the ACEA FP were good, and those of the ACEA CT were very good. The Tönnis angle was weakly correlated with inconsistent ACEA FP measurements (*P* = 0.008) but not with inconsistent ACEA CT measurements (*P* = 0.600). No correlation between ACEA FP and ACEA CT measurements was observed (*P* = 0.213–0.665). The reproducibility of the ACEA CT is more consistent than that of the ACEA FP. The oblique acetabular roof had an effect on determining the ACEA FP but not on determining the ACEA CT. No correlation was observed between the measured ACEA FP and ACEA CT values, so the clinical evidence obtained from the ACEA FP cannot be directly applied to the ACEA CT.

## BACKGROUND

Hip dysplasia occurs when the acetabulum does not cover the femoral head sufficiently, causing hip instability and pain. Traditionally, hip dysplasia has been diagnosed by determining the lateral coverage of the acetabulum to the femoral head on a pelvic anteroposterior (AP) radiograph [1]. With the improving understanding of the three-dimensional structure of the hip joint, it has been gradually realized that the cause of hip instability is a multiplanar issue encompassing all three dimensions [2, 3]. Anterior coverage is as important as lateral coverage in assessing the instability of hips [4, 5].

The anterior center-edge angle on false-profile radiography (ACEA FP) is the earliest and most commonly used parameter of anterior hip joint coverage. In 1961, Professor Lequesne began to measure the anterior center-edge angle (ACEA) on false-profile (FP) radiographs [6]. The reference value of the ACEA FP is defined as 20–40° and is set to be the target of hip correction. Good postoperative ACEA FP is significantly related to good clinical results [7].

This method also has potential problems in clinical practice. First, FP radiographs should be taken with the patient rotated accurately 65°, which is demanding for radiologists and imaging physicians. Second, for hip joints with poor lateral coverage, the anterior edge of the acetabulum on FP radiographs will be very blurred. ACEA can therefore be difficult to measure on such FP radiographs.

With the widespread application of computed tomography (CT), CT data are increasingly being used to assess the morphology of hips. CT can depict the three-dimensional structure of the hip joint, and most measurements can be taken from a single set of data, so they can replace many plain X-ray radiographs. In addition, the anterior edge of the acetabulum is clearly visible on the CT sagittal plane, so the reproducibility is higher. Hence, some scholars have begun to try to measure ACEA on CT (ACEA CT) sagittal radiographs. It was believed that only the ACEA CT sagittal reconstruction could indicate the real anterior coverage of the hip [8]. At present, there is no well-accepted reference value for the ACEA CT, and the relationship between the ACEA CT and long-term results has not yet been proven [9].

There are many differences between the ACEA when measured on the CT sagittal plane versus on FP radiographs. The ACEA CT sagittal plane measures the anterior coverage of the hip joint, while ACEA FP radiographs measure the anterolateral coverage of the hip joint. The ACEA CT is measured on only one sagittal slice, while the ACEA FP is measured through a superposition of many planar images. The ACEA CT is measured on the CT sagittal plane, which is obtained in the supine position, while the ACEA FP is measured on FP radiographs, which are obtained in the standing position. These variations lead to differences in the measurement results [10, 11].

Some studies have found that the correlation between these two parameters is poor in the normal population. To the best of our knowledge, no studies have focused on the correlation between these two parameters in patients with hip dysplasia [12].

This study aims to answer the following questions: In patients with hip dysplasia, (i) what is the interobserver and intraobserver reliability of the ACEA FP? (ii) What is the interobserver and intraobserver reliability of the ACEA CT? (iii) Does the Tönnis angle affect the measurement accuracy of the ACEA FP and ACEA CT? (iv) How relevant is the ACEA CT to the ACEA FP? (v) If there is a correlation, can the corresponding relationship be identified?

## PATIENTS AND METHODS

After approval from the Institutional Review Board, we reviewed all patients on whom isolated unilateral periacetabular osteotomy was performed for hip dysplasia in our center from July 2020 to October 2020. All 60 patients (60 hips) were followed up for over 6 months.

In these 60 cases, 7 cases (7 hips) were excluded due to severe hip deformity, which may result in inaccurate measurements. Of the seven cases, one patient had multiple epiphyseal dysplasia, two patients had severe coxa plana, where the femoral heads could not be measured, one patient had severe hip joint deformation subluxation caused by poliomyelitis and three patients had severe hip subluxation due to cerebral palsy. To determine joint congruency and femoral torsion, CT scans are routinely performed in patients prepared for periacetabular osteotomy in our center. Among the cases in this series, four hips in four cases had received CT scans at another hospital prior to hospitalization. Due to considerations of radiation exposure, CT examination was not performed again in our hospital. Their data in digital imaging and communications in medicine (DICOM) format were not available, so ACEA CT measurements could not be performed. Finally, complete radiographic data of 49 cases (49 hips) were obtained. Finally, 49 hips were included in this study.

Among the 49 patients, 44 were female, and 5 were male, with a mean age of 29.69 ± 8.59 (14–46) years at the time of surgery.

All measurements were performed on the affected side. After hospitalization, standing AP radiographs, standard FP radiographs [7] and pelvic CT were performed for each patient. Tilt correction was not performed on any standing radiographs because standing view radiographs provide a better indication of hip function with weight bearing.

Lateral center-edge angles (LCEAs) and Tönnis angles were measured on AP radiographs of the pelvis by the RadiAnt DICOM Viewer (Medixant, Poznan, Poland). The LCEA is defined as the angle between the vertical line and the line of the pelvis passing through the center of the femoral head and the lateral edge of the acetabular sourcil. The Tönnis angle is the angle between the line connecting the medial and lateral edges of the sourcil and the horizontal line [13].

**Fig. 1.**
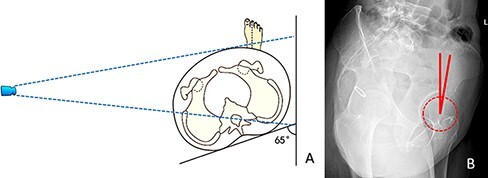
Measurement of the anterior cover of the hip joint based on the FP radiograph. (A) The filmfocus distance is 120 cm. To take a standard FP radiograph, the patient was required to rotate 65° forward to the affected side. (B) The angle between the vertical axis and the line running through the anterior edge of the acetabular sourcil and the center of the femoral head is called the ACEA FP.

The ACEA FP is obtained with an FP radiograph (Fig. 1A). It is defined as the angle between the vertical axis and the line running through the anterior edge of the acetabular sourcil and the center of the femoral head. The normal reference angle of ACEA FP is 20–40° [7] (Fig. 1B).

**Fig. 2.**
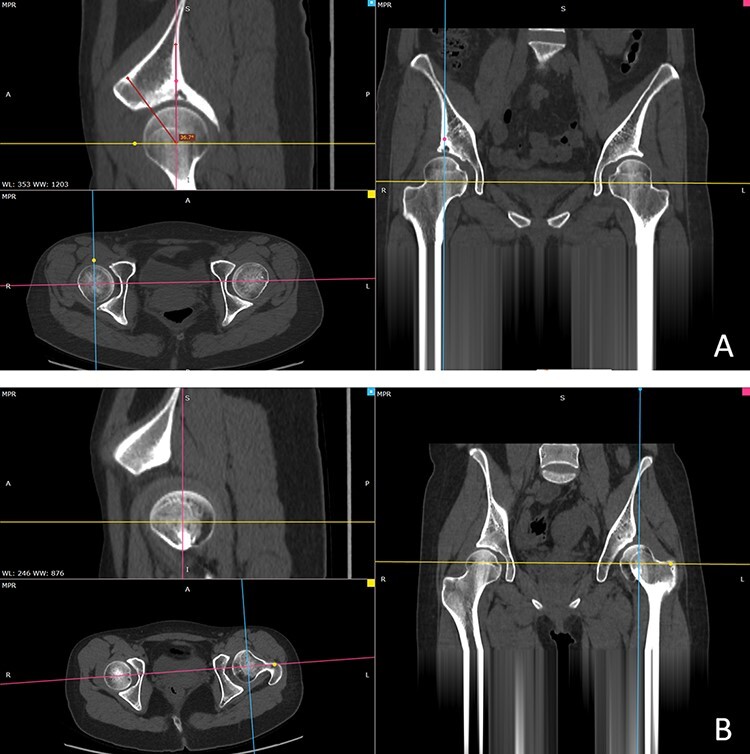
Measurement of the ACEA CT. (A) Adjust the observation plane in the multiplanar reconstruction interface to make the coronal and horizontal planes passing through the centers of both femoral heads. On the corrected sagittal plane, the angle between the vertical line and the line running through the femoral head and the anterior edge of the acetabulum is defined as the ACEA CT. (B) When the LCEA was less than zero, the acetabulum did not cover the femoral head on the sagittal plane, which goes through the center of the femoral head.

A dense CT scan of the pelvis was performed preoperatively. The CT interval was 1.3 mm. The RadiAnt DICOM Viewer was used to conduct multiplanar reconstructions. If the pelvis is inclined, the observation plane should be adjusted at the multiplanar reconstruction interface to make the coronal and horizontal planes passing through the center of both femoral heads. The ACEA CT was measured on the sagittal surface by placing the angle of the line vertically up through the center of the femoral head and the line between the femoral head and the anterior edge of the acetabulum on the adjusted sagittal surface (Fig. 2A) [9]. If the distance between the acetabulum and the femoral head was more than 5 mm, the three views were reviewed to ensure that there was a matching articular surface. Generally, when the LCEA is less than zero, the femoral head is not covered by the acetabulum on the sagittal plane, which goes through the center of the femoral head, so it cannot be measured. Although these measurements are defined as 0 in some studies, this does not truly indicate the real anterior coverage of the femoral head (Fig. 2B) [14]. For these patients, valid data could not be obtained by this method. Therefore, these data were not included in our statistical analysis.

Both observers (Observer A and Observer B) were experienced hip surgeons who were able to perform periacetabular osteotomy independently. These values were measured by each observer twice, 4 weeks apart.

### Statistical methods

Statistical product and service solutions, (SPSS) 26 (IBM, Armonk, NY, USA) software was used for statistical analysis. All data were tested by the Shapiro–Wilk test to check whether the data had a normal distribution. The mean value and standard deviation were calculated for all data of the two participants in the second measurement. The interclass correlation coefficient (ICC) was analyzed between two measurements of the same indicator and the same observer. Absolute agreement was calculated using the two-way random model. ICC analysis was carried out on measurements taken between different observers of the same parameter at the same time to observe their absolute agreement with a two-way random model. Agreement was interpreted as follows: poor, ICC < 0.20; fair, ICC = 0.21–0.40; moderate, ICC = 0.41–0.60; good, ICC = 0.61–0.80 and very good, ICC > 0.80.

The variance between the four ACEA FPs for every patient (measured by two observers two times) was determined. The same was performed for the ACEA CTs. The correlation between the intraobserver and interobserver inconsistency and the Tönnis angle was observed by evaluating the correlation between the Tönnis angle and the variance in ACEA FP and between the Tönnis angle and the variance in the ACEA CT.

Correlation analysis was conducted between the mean of all ACEA FPs measured by each observer and the corresponding mean of the ACEA CT to observe their correlation, and the correlation between every ACEA FP value measured by each observer each time and the corresponding ACEA CT values was analyzed. In all the above cases, Pearson’s correlation was used when data showed a normal distribution, and Spearman’s correlation was used when data did not show a normal distribution. The *R* value was interpreted as follows: very weak, *R* = 0–0.19; weak, *R* = 0.20–0.39; moderate, *R* = 0.40–0.59; strong, *R* = 0.60–0.79 and very strong, *R* = 0.80–1.0.

## RESULTS

Data were obtained for 49 hips in 49 patients. Because the lateral edge of the acetabulum did not reach the center of the femoral head, the anterior coverage of the acetabulum to the femoral head could not be measured on the sagittal plane of the femoral head center. Data from seven hips in seven cases were not included in the statistical analysis. All data, including the ACEA FP, ACEA CT and Tönnis angle, from our 42 hips in 42 cases that we statistically analyzed were normally distributed.

The mean Tönnis angle on pelvic AP radiographs was 24.2 ± 6.1 (12.1–36.6).

The ACEA FP from Observer A was 11.0 ± 13.5 (−21.4 to 37.0) for the first time and 13.8 ± 14.0 (−17.4 to 40.9) for the second time. The ACEA FP from Observer B was 7.9 ± 14.3 (−25.8 to 34.0) for the first time and 6.6 ± 14.3 (−23.5 to 35.4) for the second time (Table I).

**Table I.** Mean value, standard deviation and reliability of the ACEA FP and ACEA CT

*Parameters*	*Mean ± SD*	*Intraobserver ICC of Observer A*	*Intraobserver ICC of Observer B*	*Interobserver ICC for the first time*	*Interobserver ICC for the second time*
ACEA FP	9.8 ± 12.4	0.783	0.779	0.793	0.710
ACEA CT	42.6 ± 10.2	0.952	0.913	0.802	0.860

The ACEA CT from Observer A was 42.5 ± 11.9 (8.7–63.8) for the first time and 41.9 ± 11.9 (8.4–65.9) for the second time. The ACEA CT from Observer B was 42.9 ± 9.3 (13.3–62.1) for the first time and 43.2 ± 9.6 (15.1–63.4) for the second time (Table I).

For each subject, the ACEA FP was measured four times by two observers. The variance in these four measurements for every subject did not fit a normal distribution. There was a weak correlation between the Tönnis angle and the variance in the ACEA FP (*P* = 0.008, correlation coefficient 0.374).

The variance in the four ACEA CT measurements for every subject also did not fit a normal distribution. There was no correlation between the Tönnis angle and the variance in the ACEA CT (*P* = 0.600).

**Fig. 3.**
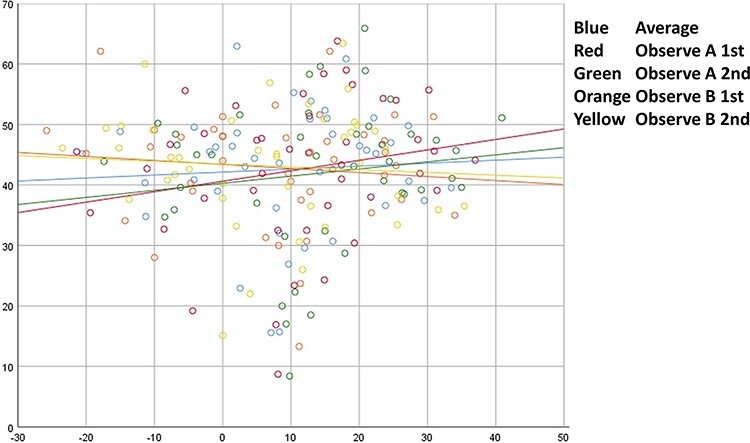
In the figure, red indicates the first measurement of Observer A, green indicates the second measurement of Observer A, orange indicates the first measurement of Observer B and yellow indicates the second measurement of Observer B. Blue is the average of the four measurements.

In the four measurements from two observers, the ACEA FP and ACEA CT measured by each observer were not correlated (Observer A, first time *P* = 0.213; Observer A, second time *P* = 0.383; Observer B, first time *P* = 0.523; Observer B, second time *P* = 0.665). There was also no correlation between the mean ACEA FP and the mean ACEA CT (*P* = 0.707), as shown in Fig. 3.

## DISCUSSION

Inadequate anterior coverage could cause hip instability and eventually lead to hip pain and early osteoarthritis. The ACEA FP is the most commonly used parameter of anterior coverage in existing studies. Since the measurement of the ACEA FP is based on the identification of the anterior edge of the acetabular sourcil, it is difficult to perform when the sourcil is blurred. With the widespread use of CT, the ACEA CT has begun to be used to determine the anterior coverage of the hip instead of the ACEA FP. The reproducibility of the ACEA CT is better, but its correlation with the ACEA FP in patients with hip dysplasia is not yet clear [10]. To address these problems, we carried out this study.

First, the intraobserver and interobserver reliability of the ACEA FP and ACEA CT used in dysplastic hips were observed. We found that in patients with hip dysplasia, both the intraobserver and interobserver reliability of the ACEA FP were good. The intraobserver and interobserver reliability of the ACEA CT were significantly higher than those of the ACEA FP, both of which are very good. Because the sagittal plane of CT shows the anterior edge of the acetabulum more clearly, the problem of anterior edge identification is resolved. Both the interobserver and intraobserver reliability were higher, which are consistent with our assumption.

**Fig. 4.**
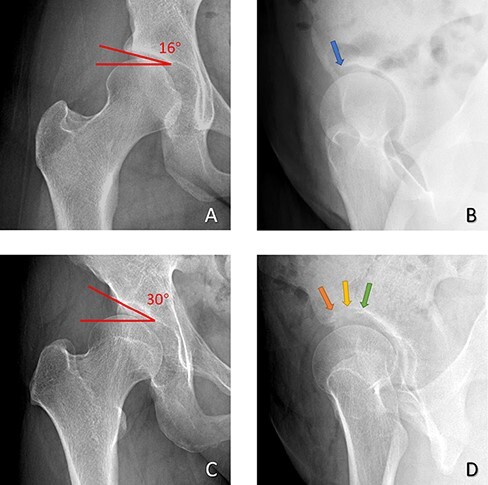
The relationship between the Tönnis angle (Red) and the clarity of sourcil on FP radiography. (A) The Tönnis angle of Case 1 is 16° on AP radiography. (B) The acetabular anterior edge of Case 1 can be clearly identified (blue arrow). The measurement reproducibility of Case 1 is good. (C) The Tönnis angle of Case 2 is 30° on AP radiography. (D) The sourcil of Case 2 on FP radiography is blurred. The acetabular anterior edge was identified as a different site (orange, yellow and green arrows) in different measurements by different observers.

Compared with previous studies performed on the normal pelvis [15], in the present study of patients with dysplasia, the intraobserver and interobserver correlations were lower. This is consistent with our clinical experience that the more severe the hip dysplasia, the more difficult it can be to measure the anterior coverage. When we further evaluated the relationship between the Tönnis angle and the accuracy of the ACEA FP and ACEA CT, we found that the measurement variety of the ACEA FP increased when the Tönnis angle was larger. However, no obvious relation was found between the Tönnis angle and the accuracy of the ACEA CT. The measurement differences mainly result from the determination of the anterior edge of the acetabular sourcil. The acetabular sourcil is formed by the overlayed projection of acetabular subcartilaginous bones on FP radiographs. When the sourcil is almost horizontal on AP radiography, the subcartilaginous bones can be better presented on FP radiography, showing a clearer ‘sourcil’ (Fig. 4A and B). In patients with severer hip dysplasia, as the Tönnis angle increased on AP radiography, the subcartilaginous bones could not overlap enough to form a clear sourcil. The blurred sourcil makes the anterior edge of the acetabulum difficult to identify, which results in an increased discrepancy between observations (Fig. 4C and D).

The effect of the Tönnis angle on the ACEA CT was also assessed in this study, but no correlation was found. However, in contrast to the ACEA FP, the ACEA CT measures one CT slice instead of overlapping images. The anterior acetabular edge is clearly discernible on CT images, regardless of whether the acetabular roof was horizontal. Therefore, oblique acetabular roof has no influence on CT observation.

Surprisingly, the measured values of the ACEA FP and ACEA CT, both of which are parameters of anterior hip coverage, were assumed to be correlated, but no correlation was observed (*P* = 0.707). This is in line with the previous study on normal hips [15]. Although both ACEA FP and ACEA CT are parameters of acetabular hip coverage, there are many differences in measurement methods. The ACEA FP is measured on FP radiography, which is formed by projection, while the ACEA CT is measured on only one CT slice. The ACEA FP revealed anterolateral coverage of the acetabulum at the 65° rotated position [10], while the ACEA CT revealed anterior coverage of the acetabulum on the sagittal plane. The ACEA FP is measured when patients are standing, while the ACEA CT is measured when patients are in the supine position. Because the tilt of the pelvis may affect the measurement of anterior hip coverage, [16, 17] no correlation was found between the ACEA FP and ACEA CT.

We cannot simply use anterior hip coverage, which was measured by the ACEA CT, to evaluate clinical results when the clinical relevance got from ACEA FP. Based on their different characteristics, ACEA FP could be more suitable for determining anterior hip stability [18–20] because ACEA FP was taken in a standing position. That is the position in which the patient’s hip joint is actually performing its weight-bearing function [21]. While ACEA CT describes the morphology of the anterior hip joint more precisely, it is more suitable for evaluating the potential impingement which may occur after hip reorientation [19, 22–25].

There are some limitations to this study:

- In patients with severe hip dysplasia, when the LCEA is less than 0°, the acetabulum is often unable to contact the femoral head on the sagittal plane through the center of the femoral head [23]. In these cases, the ACEA CT is not available. However, some studies have used 0 to represent the anterior hip coverage in this situation [24]. Because it is not very meaningful to consider the anterior coverage before lateral coverage correction, these patients were excluded from the study, in which we focused on anterior coverage.
- The acetabulum is a three-dimensional structure. The anterior coverage proposed in this paper is still discussed based on the previous two-dimensional indexes instead of real three-dimensional indexes. The first reason is that all previous studies on the correlation between anterior hip coverage and long-term result use two-dimensional parameters. To the best of our knowledge, there is no direct clinical proof that three-dimensional measures of anterior hip coverage would influence the results. The second reason is that the current three-dimensional data are all from CT, which is obtained with the patient in the supine position, so the patient’s weight-bearing position cannot be accurately reproduced.

Although the hip joint is a three-dimensional structure, two-dimensional parameters cannot accurately describe its three-dimensional structure, the ACEA FP that can be used as a surrogate parameter of anterior hip coverage for patients with hip dysplasia can predict the long-term prognosis of the hip because of existing clinical evidence. The way we can obtain an accurate ACEA FP from a severely deficient acetabulum is to evaluate the anterior edge of the sourcil with great care. In this study, there tended to be no correlation between the ACEA CT and ACEA FP. Since both the ACEA FP and ACEA CT are anterior coverage parameters, they do not describe the same anterior hip coverage. It is hasty to use the results of each other’s clinical trials to estimate the prognosis. The correlation between the ACEA CT and clinical prognosis remains questionable and should be confirmed in the future.

## Data Availability

Data are available upon request.
